# Preventing Complex Regional Pain Syndrome After Distal Radius Fracture: A Systematic Review of Rehabilitation and Clinical Prophylaxis Strategies

**DOI:** 10.3390/jfmk11020158

**Published:** 2026-04-17

**Authors:** Inês Neves Serôdio, Olalla Saiz-Vázquez, Hilario Ortiz-Huerta, Lucia Simón-Vicente, Montserrat Santamaría-Vázquez

**Affiliations:** 1 School of Health Sciences, Polytechnic of Leiria, Campus 2-Morro do Lena, Alto do Vieiro-Apartado 4137, 2411-901 Leiria, Portugal; ines.n.serodio@ipleiria.pt; 2 Hospital de Faro–Centro Hospitalar e Universitario do Algarve, R. Leão Penedo, 8000-386 Faro, Portugal; 3 Health Sciences Department, Universidad de Burgos, Paseo Comendadores s/n, 09001 Burgos, Spain

**Keywords:** complex regional pain syndrome, distal radius fracture, prevention, rehabilitation, home exercise program, vitamin C, systematic review

## Abstract

**Background**: Complex regional pain syndrome (CRPS) is a disabling post-traumatic pain condition that may occur after distal radius fracture (DRF), potentially impairing recovery and upper-limb function. Identifying effective preventive strategies after DRF is therefore clinically important. **Objective**: To synthesize and critically appraise interventions intended to prevent CRPS after DRF, including rehabilitation protocols and clinical prophylaxis strategies. **Methods**: This systematic review followed Preferred Reporting Items for Systematic Reviews and Meta-Analyses PRISMA and was registered in the International Prospective Register of Systematic Reviews PROSPERO (CRD42023408499). Five databases (PubMed, Web of Science, Scopus, ScienceDirect, and B-on) were searched for studies published from January 2013 to 22 September 2023 in English, Portuguese, or Spanish. The primary outcome was CRPS incidence after DRF. Findings were synthesized narratively due to heterogeneity in interventions and diagnostic criteria, and risk of bias was assessed using design-appropriate tools. **Results**: Nine studies were included (total N = 7075; CRPS cases n = 127). Interventions comprised vitamin C supplementation (2 studies), probiotics, aspirin, polarized/polychromatic light therapy plus conventional treatment, early rehabilitation/home-exercise programs, and general CRPS-prevention protocols after DRF. Probiotics and aspirin did not reduce CRPS incidence. Vitamin C showed mixed findings across the included studies and remains debated in the broader literature. Light therapy was associated with reduced CRPS occurrence in a single study, while early active home-exercise programs appeared promising but were supported by a limited number of studies. Study designs and CRPS diagnostic criteria varied, and risk of bias was moderate-to-serious in several non-randomized studies. **Conclusions**: Evidence remains insufficient to support a single standardized prevention protocol for CRPS after distal radius fracture. Early active rehabilitation and progressive mobilization appear promising, but the available evidence is still limited and heterogeneous. Adjunctive strategies such as vitamin C and light therapy should be interpreted with caution, as findings for vitamin C remain debated in the literature and the evidence for light therapy is currently based on a single study. Other approaches, including probiotics and aspirin, have shown inconclusive results.

## 1. Introduction

Complex Regional Pain Syndrome (CRPS) is a chronic and often disabling pain disorder that typically develops after trauma, including fractures and surgical procedures [1]. It is characterized by continuous, disproportionate pain accompanied by sensory, vasomotor, sudomotor/edema, and motor/trophic abnormalities [2]. CRPS is currently classified into three diagnostic subtypes: CRPS type I, in which no major nerve injury is identified; CRPS type II, in which a distinct nerve injury is present; and CRPS-not otherwise specified (CRPS-NOS), which applies to patients whose clinical presentation is consistent with CRPS but does not fully meet the established diagnostic criteria or for whom insufficient information is available. Despite this classification, CRPS type I and type II share a largely similar clinical phenotype involving sensory, vasomotor, sudomotor/edema, and motor/trophic disturbances [3,4,5].

Accurate diagnosis is difficult because there is no definitive confirmatory test for CRPS. The condition is diagnosed primarily based on clinical history and physical examination findings [6]. To improve diagnostic accuracy and facilitate research, the Budapest Criteria were developed and later endorsed by the International Association for the Study of Pain (IASP), as well as incorporated into the Royal College of Physicians (RCP) and British Orthopaedic Association (BOA) guidelines [7]. These criteria provide a structured diagnostic framework based on four domains (sensory, vasomotor, sudomotor/edema, and motor/trophic). However, despite their widespread acceptance, variability in diagnostic approach persists in routine clinical practice. This may contribute to delayed recognition and to uncertainty regarding the true epidemiological burden of CRPS, particularly because reported incidence estimates vary according to the diagnostic criteria applied [7,8,9].

CRPS has a variable clinical course, with recovery rates estimated at 74% within the first year but declining substantially over time, with only 36% achieving meaningful resolution six years after onset [1]. Its impact on quality of life is substantial due to persistent pain, motor dysfunction, emotional distress, and limitations in daily functioning [2]. Psychological complications such as anxiety, kinesiophobia, and depression may further intensify disability [2].

Epidemiological data suggest that CRPS is more common in specific demographic groups. Individuals between 55 and 75 years of age show the highest incidence, with risk increasing steadily until age 70 [10]. The condition is significantly more prevalent in women, who have a 3.5-fold increased likelihood of developing CRPS compared with men [11]. Although CRPS may occur in response to various injuries, distal radius fracture (DRF) constitutes the single most common precipitating event, and represents the primary trigger for CRPS in multiple studies [12]. Reported CRPS incidence following DRF varies widely (from 1% to 37%) depending on diagnostic criteria, treatment method, and population characteristics [12].

Given its high functional and psychological burden, prevention of CRPS after DRF has become a priority in hand surgery, rehabilitation, and pain medicine. Numerous risk factors have been associated with post-DRF CRPS, including female sex, low- to medium-energy trauma, immobilization with casting, motor nerve injury, advanced age, fibromyalgia, rheumatoid arthritis, open fractures, psychiatric comorbidities, and high levels of acute post-injury pain [13]. Many of these factors are non-modifiable, but some (such as immobilization practices, early mobilization, pain management, and patient education) may be targeted clinically to reduce the likelihood of developing CRPS.

Despite growing interest in prevention, no consensus exists regarding the most effective strategies to reduce CRPS incidence after DRF. Proposed interventions include vitamin C, aspirin, probiotics, light therapy, and early rehabilitation programs. However, the available evidence remains inconsistent and is limited by methodological heterogeneity, variable diagnostic criteria, and small sample sizes, making it difficult to identify reliable preventive measures for clinical practice.

These gaps highlight the need for an updated systematic evaluation of preventive interventions for CRPS after distal radius fracture. The primary aim of this systematic review was to assess the effectiveness of the available preventive strategies. The secondary aim was to evaluate the methodological quality of the included studies and to consider the clinical applicability of these interventions in rehabilitation practice.

## 2. Materials and Methods

This systematic review was conducted in accordance with the Preferred Reporting Items for Systematic Reviews and Meta-Analyses (PRISMA) guidelines [14]. The protocol was prospectively registered in PROSPERO on 15 March 2023 (Ref. CRD42023408499). The review focused on identifying interventions aimed at preventing Complex Regional Pain Syndrome (CRPS) following Distal Radius Fracture (DRF).

### 2.1. Search Strategy

A comprehensive literature search was carried out in five electronic databases: PubMed, Web of Science, Scopus, ScienceDirect and B-on. Searches included articles published between January 2013 and 22 September 2023, in English, Portuguese, or Spanish. This time window was selected to provide an updated synthesis of contemporary evidence on preventive interventions for CRPS after distal radius fracture, with greater applicability to current clinical practice and more recent diagnostic frameworks. Search terms combined controlled vocabulary and free-text words related to CRPS and its historical synonyms, as well as terms related to prevention and distal radius fracture. The PubMed search strategy was as follows: ((“complex regional pain syndrome” [Title/Abstract]) OR (“reflex sympathetic dystrophy” [Title/Abstract]) OR causalgia [Title/Abstract] OR algodystrophy [Title/Abstract] OR Sudeck [Title/Abstract] OR (“post-traumatic dystrophy” [Title/Abstract])) AND ((prevention [Title/Abstract]) OR prophylaxis [Title/Abstract] OR preventive [Title/Abstract]) AND ((“distal radius fracture” [Title/Abstract]) OR (“wrist fracture” [Title/Abstract])). This strategy was adapted to the syntax and indexing system of each database using equivalent Boolean operators and controlled vocabulary where available.

### 2.2. Eligibility Criteria

As this review synthesized evidence from published studies rather than recruiting participants from specific clinical centers, eligibility criteria were applied to the identified studies and not to participating institutions. Studies were considered eligible if they included adult participants with a distal radius fracture and evaluated any intervention specifically intended to prevent Complex Regional Pain Syndrome (CRPS), such as rehabilitation approaches, pharmacological or nutritional prophylaxis, or other clinical or physical strategies. To be included, studies needed to report CRPS incidence using recognized diagnostic criteria or clear clinical description and be designed as randomized controlled trials, prospective or retrospective cohort studies, or controlled clinical studies. Only articles published between 2013 and 22 September 2023, in English, Portuguese, or Spanish were included. Studies were excluded if they were editorials, letters, technical notes, preclinical investigations, books, documents, review articles, meta-analyses, or if they did not describe a preventive intervention for CRPS.

### 2.3. Study Selection

After removing duplicates, two reviewers independently screened titles and abstracts for relevance. Full texts of potentially eligible studies were then assessed in detail according to the predefined eligibility criteria. Discrepancies were resolved through discussion, and when necessary, a third reviewer contributed to consensus.

**Figure 1:**
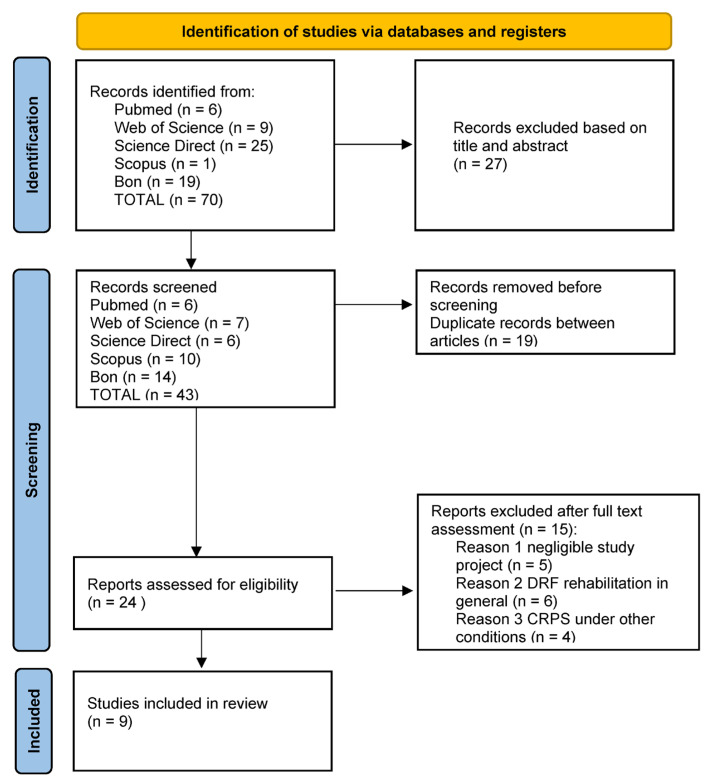
PRISMA 2020 flow diagram of study selection (identification, screening, eligibility, and included studies).

A PRISMA flow diagram was generated to document the number of records identified, screened, excluded, and included (Figure 1).

### 2.4. Data Extraction

Data extraction was carried out independently by two reviewers using a standardized spreadsheet to ensure consistency and accuracy. For each included study, the reviewers collected information on authorship, year of publication, study design, sample size, participant characteristics, diagnostic criteria used to identify CRPS, type of preventive intervention employed, comparator groups, duration of follow-up, and the reported incidence of CRPS. Key findings relevant to the prevention of CRPS were also extracted. Any discrepancies in the extracted data were discussed and resolved through consensus to ensure the reliability of the final dataset.

### 2.5. Risk of Bias Assessment

**Figure 2:**
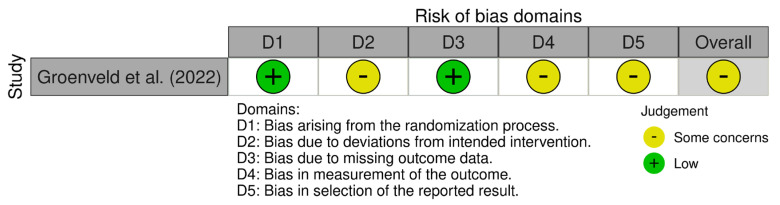
Risk of bias assessment for randomized trials using the revised Cochrane Risk of Bias tool for randomized trials (RoB 2) [17].

Risk of bias was assessed at the study level for the primary outcome (CRPS incidence) using design-specific tools. The randomized controlled trial was appraised with the revised Cochrane Risk of Bias tool [15] (RoB 2) (Figure 2), evaluating bias arising from the randomization process (D1), deviations from intended interventions (D2), missing outcome data (D3), measurement of the outcome (D4), and selection of the reported result (D5), with an overall judgement of low risk, some concerns, or high risk.

**Figure 3:**
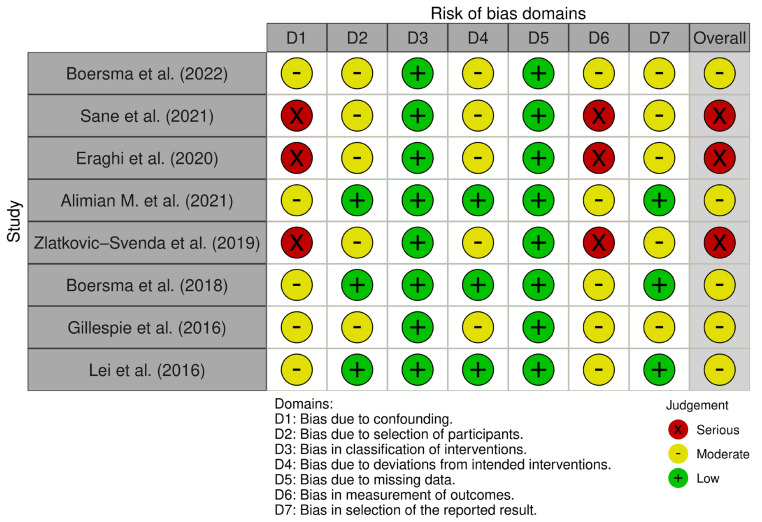
Risk of bias assessment for non-randomized studies using ROBINS-E (Risk Of Bias In Non-randomized Studies—of Interventions) [11,12,18,19,20,21,22,23].

Non-randomized studies of interventions were assessed with the ROBINS-E tool [16] (Figure 3), addressing bias due to confounding (D1), selection of participants (D2), classification of interventions (D3), deviations from intended interventions (D4), missing data (D5), measurement of outcomes (D6), and selection of the reported result (D7). Overall judgements were categorized as low, moderate, serious, or critical risk of bias. Results were summarized using domain-level and overall “traffic-light” visualizations. Risk of bias was assessed independently by two reviewers using RoB 2 for randomized trials and ROBINS-E for non-randomized studies. Any disagreements were resolved by consensus.

### 2.6. Certainty of Evidence (Adapted GRADE Approach)

The certainty of evidence for the primary outcome (CRPS incidence after distal radius fracture) and relevant secondary outcomes (pain and upper-limb function/disability when reported) was assessed using the (Adapted Grading of Recommendations Assessment, Development and Evaluation (GRADE) Approach) approach. For each intervention category, evidence was initially rated as high (randomized trials) or low (non-randomized studies) and then downgraded, when appropriate, for risk of bias, inconsistency, indirectness, imprecision, and publication bias. A Summary of Findings table was produced to present effect direction/magnitude and the final certainty rating for each comparison. The GRADE Summary of Findings table is provided in Supplementary Table S1.

### 2.7. Data Synthesis

Given the heterogeneity in study designs, interventions, diagnostic criteria, and outcome definitions, a meta-analysis was not feasible. Therefore, a narrative synthesis approach was used to summarize findings across studies, structured by type of preventive intervention. Due to clinical and methodological heterogeneity (interventions, outcome definitions, and CRPS diagnostic criteria), a meta-analysis was not performed; results were synthesized narratively and structured by intervention category.

## 3. Results

The study identified 70 studies, with 46 included after excluding duplicates and articles revised by title and abstract. The full text of each selected article was screened to ensure it met all criteria, resulting in 9 studies for further analysis. Figure 1 shows the study selection process and the number of excluded articles in each phase.

The characteristics of the included studies are described in Table 1. The total number of participants in this review is N = 7075, of which n = 127 were diagnosed with CRPS, despite the preventive measures proposed by the authors. The average age of participants ranged from 45 to 89 years. The CRPS diagnosis was based on one of the following criteria: Budapest Criteria [24] (N = 4); Atkins Criteria [25] (N = 1); Bruehl’s Diagnostic Criteria [26] (N = 1); Veldman Criteria [27] (N = 1) or on non-validated clinical criteria (N = 2).

The systematic review identified five preventive measures for CRPS in DRF patients: Vitamin C, probiotics, aspirin, polarized light therapy, conventional treatment, and early rehabilitation program.

### 3.1. Vitamin C

Two studies (n = 2) have investigated the effectiveness of vitamin C in preventing CRPS after a fracture repair (DRF). Sane et al. [12] and Alimian M. et al. [20] used two groups with unilateral DRF, each receiving standard therapy, post-treatment oral calcium supplementation, and vitamin D for three months. Both groups also started taking oral vitamin C. The results showed that the overall incidence of CRPS at follow-up in vitamin C groups was significantly less than the controls, with an overall incidence of 11.3% against 26% and 22.9% against 45.5%, respectively.

### 3.2. Probiotics

M. Lei et al. [23] studied the impact of probiotic treatment on functional recovery in elderly patients with a distal radius fracture (DRF). They used a randomized sample of patients who received skimmed milk containing either a commercial probiotic (*Lactobacillus casei* Shirota-LcS) or a placebo for 6 months. The LcS group had significantly lower CRPS scores in the first three months, but at month 4, it was still lower. The study concluded that LcS could accelerate DRF healing and improve functional recovery.

### 3.3. Aspirin

Eraghi A.S. et al. [19] evaluated the effects of aspirin in prevention of the CRPS following a DRF in an experimental group randomly allocated to receive either placebo or 500 mg of aspirin (ASA) daily for 7 days. The prevalence of CRPS in the aspirin group was lower (13.6%) than the placebo group (19.1%), but the difference was not statistically significant.

### 3.4. Polarised, Polychromatic Light Therapy

A study by Zlatkovic-Svenda et al. [21] investigated the use of polarised, polychromatic, low-energy light therapy combined with conventional treatment with DRF after plaster removal in elderly people. The study involved two groups: one treated with exercises and cryotherapy, and the other with Bioptron light therapy. The light therapy group received 10 min of Bioptron light therapy daily on five points of the dorsal hand region for 15 days. The light therapy-treated group significantly reduced CRPS occurrence compared to the control group.

### 3.5. Rehabilitation Program

Boersma’s studies [11,18] investigated the potential of early activity post-injury to prevent CRPS in cases of DRF. The first study applied a home exercise program after cast removal to patients with DRF, teaching them to exercise their fingers, forearm, and wrist. Patients were allowed to resume normal activities, gradually increasing in weight and force. The second study used a similar protocol in Nijmegen, The Netherlands, but between December 2012 and July 2017, none of the patients were diagnosed with CRPS-I, resulting in an incidence of 0%. Both studies showed promising results in preventing CRPS in DRF patients.

### 3.6. Incidence Studies

Groenveld et al. [17] studied the incidence of complex regional pain syndrome (CRPS) after distal radius fracture (DRF) in the Netherlands from 2014 to 2018, finding a 0.36% decrease in annual incidence. They suggested that the change in clinical approach towards CRPS, focusing on prevention and psychological aspects, may be a factor in this decrease. Gillespie et al. [22] conducted audits and service evaluations at the Royal Liverpool University Hospital (RLBUHT) between 2004 and 2013, finding a 25% incidence of post-DRF CRPS in 2004 and a 0.6% reduction in 2013. They concluded that the incidence dropped to 10% and linked it to audits, patient information programs, staff education, and a patient tolerance culture.

### 3.7. Overall Summary of the Included Studies

Table 2 presents preventive protocols for complex regional pain syndrome (CRPS) reported in the included studies. Active approaches like rehabilitation programs and light therapy combined with conventional treatment had the best results, with 0% CRPS incidence [11,18,21]. Vitamin C supplementation showed lower CRPS incidence than comparator interventions in some studies [12,20], whereas aspirin did not demonstrate a significant preventive effect and probiotics showed no clear benefit [19]. Interpretation of CRPS incidence across studies was limited by variability in diagnostic criteria and incomplete reporting in some articles [23]. However, changes in clinical approach, such as shorter immobilization periods, differential diagnosis, informed patient care, and active home exercise programs, can reduce CRPS incidence [17,22].

## 4. Discussion

The present systematic review synthesizes the available evidence on interventions aimed at preventing Complex Regional Pain Syndrome (CRPS) following distal radius fracture (DRF). Given the growing incidence of DRF worldwide (estimated at 228 per 100,000 individuals per year and increasing by approximately 20% over the last two decades) [29], understanding strategies that may reduce the risk of CRPS is of major clinical importance.

In recent years, considerable attention has been directed toward early identification of risk factors and potential preventive measures for CRPS after DRF [6,13]. Among the studied interventions, vitamin C has received the most extensive investigation (initially proposed by Zollinger in 1999 [30] and subsequently explored in several reviews [31,32] and a meta-analysis [33]). The findings of the present review are consistent with that body of literature. Most included studies suggest that vitamin C supplementation (commonly administered at doses of 500 to 1000 mg daily for 6 to 12 weeks) may reduce CRPS incidence in patients with DRF [11,20,31,33]. These effects have been attributed to vitamin C’s antioxidant properties and its potential role in mitigating local inflammatory cascades involved in CRPS pathophysiology. However, not all studies have reported beneficial effects. Ekrol et al. [34], included in earlier reviews [32], found no significant long-term reduction in CRPS prevalence and even reported a higher short-term incidence in non-displaced fractures among patients receiving vitamin C. These inconsistencies underscore the need for further high-quality trials to determine optimal dosage, timing, and patient selection.

Beyond vitamin C, fewer studies have investigated alternative preventive interventions. Probiotic therapy was explored in one randomized controlled trial, likely based on the hypothesis that modulation of the gut microbiota may influence systemic inflammatory responses and potentially support tissue recovery after fracture. In that study, probiotic supplementation was associated with some early improvements in CRPS-related scores and functional recovery [23]. However, these findings remain preliminary, and the absence of consistent effects on CRPS incidence may reflect the indirect nature of the proposed mechanism, as well as the fact that probiotics may benefit general recovery without necessarily preventing the specific pathophysiological processes involved in CRPS. Similarly, aspirin was evaluated because of its anti-inflammatory and antiplatelet properties, which could theoretically reduce post-traumatic inflammatory responses associated with CRPS development. Nevertheless, the available study showed only a non-significant reduction in CRPS incidence compared with placebo [19], suggesting that short-term aspirin administration may have been insufficient in dose, duration, or mechanism to meaningfully alter CRPS risk. Overall, the limited number of studies and modest effects highlight the need for further research into pharmacological prophylaxis beyond antioxidant supplementation.

The use of polarized and polychromatic light therapy (applied alongside conventional rehabilitation) also demonstrated potential preventive effects [21]. However, this evidence is derived from a single study with specific treatment parameters, making it difficult to determine the generalizability of the findings. More robust trials are needed before such modalities can be considered beyond an exploratory option in routine post-fracture care.

Early mobilization and active rehabilitation programs appear promising as preventive strategies. Two studies included in this review reported an incidence of 0% CRPS in patients following structured home exercise programs initiated soon after cast removal [11,18]. However, the number of studies remains small, and the available data are still insufficient to support definitive conclusions regarding efficacy. These findings align with the theoretical rationale that early activity may counteract disuse, maladaptive neuroplasticity, and psychological factors associated with CRPS development. The role of patient education, expectation management, and maintenance of normal movement patterns is further supported by broader clinical observations, as studies focusing on comprehensive patient-centered care pathways have reported reductions in CRPS incidence at the population level [17,22]. Although encouraging, the number of controlled studies evaluating early rehabilitation specifically as a preventive tool remains small, highlighting a relevant gap in the literature.

Collectively, the results of this review suggest that some interventions, particularly early mobilization and selected adjunctive strategies, may have preventive potential after DRF; however, the available evidence remains limited and uneven across interventions. In particular, findings regarding vitamin C should be interpreted cautiously, as evidence remains debated in the literature despite some positive results in the studies included in this review. However, the overall evidence remains limited by heterogeneity in study designs, variability in diagnostic criteria, differences in follow-up duration, and methodological inconsistencies. The use of Budapest, Atkins, Bruehl, Veldman, or non-validated clinical criteria across studies introduces variability that may affect diagnostic accuracy and comparability of findings [24,25,26,27]. Additionally, differences in immobilization protocols, surgical techniques, rehabilitation approaches, and patient characteristics complicate the interpretation of pooled results.

Although the present review focuses on adults with distal radius fracture, previous CRPS literature outside this specific population has also emphasized ongoing challenges in diagnosis and treatment. Pediatric reviews, in particular, describe CRPS management as still debated and inherently multidisciplinary, underscoring the importance of combining rehabilitation, psychological, and medical strategies. While these findings are not directly generalizable to the adult post-fracture context, they support the broader view that CRPS management benefits from early recognition and interdisciplinary care [35,36].

Only nine studies were eligible for inclusion in the final synthesis, despite the cumulative sample size of 7075 participants. Therefore, the available evidence should be interpreted with caution, as the small number of studies and their substantial clinical and methodological heterogeneity limit the robustness and generalizability of the conclusions. Another limitation of this review is the restriction of the search to studies published between 2013 and 2023. Although this decision was intended to provide an updated overview of evidence with greater applicability to current clinical practice, it may have excluded earlier high-quality studies and thus introduced selection bias. This is particularly relevant in relation to vitamin C prophylaxis, since several influential trials published before 2013 contributed substantially to the evidence base in this field but were not eligible for inclusion in the present review. Accordingly, the findings should be interpreted as a synthesis of recent evidence rather than as an exhaustive historical review of all preventive strategies for CRPS after distal radius fracture. Given these limitations, there is a clear need for rigorous, adequately powered randomized controlled trials that use standardized diagnostic criteria and clearly defined interventions. Future research should also explore cost-effectiveness, feasibility in real-world clinical settings, and the combined impact of multimodal preventive strategies (especially those integrating education, early activity, and psychological support).

This review has several strengths. It provides an updated overview of preventive strategies for CRPS after distal radius fracture, includes a broad range of interventions, and was conducted using a transparent methodology based on PRISMA guidance and prospective PROSPERO registration. In addition, independent screening and data extraction strengthened the methodological rigor of the review.

From a rehabilitation perspective, CRPS prevention after distal radius fracture should be approached as a multimodal process rather than as a single intervention. Practical measures may include early patient education, progressive home exercise or supervised rehabilitation, and regular follow-up to identify warning signs such as disproportionate pain, edema, vasomotor changes, or movement avoidance. These actions may support early recognition and timely interdisciplinary management when CRPS is suspected. Adjunctive strategies such as vitamin C, light therapy, or other pharmacological approaches may be considered on a case-by-case basis, but their role should be interpreted cautiously given the heterogeneity of the available evidence. Future studies should use standardized diagnostic criteria, clearly report rehabilitation protocols and adherence, and include functional outcomes that are directly relevant to clinical rehabilitation.

**Figure 4:**
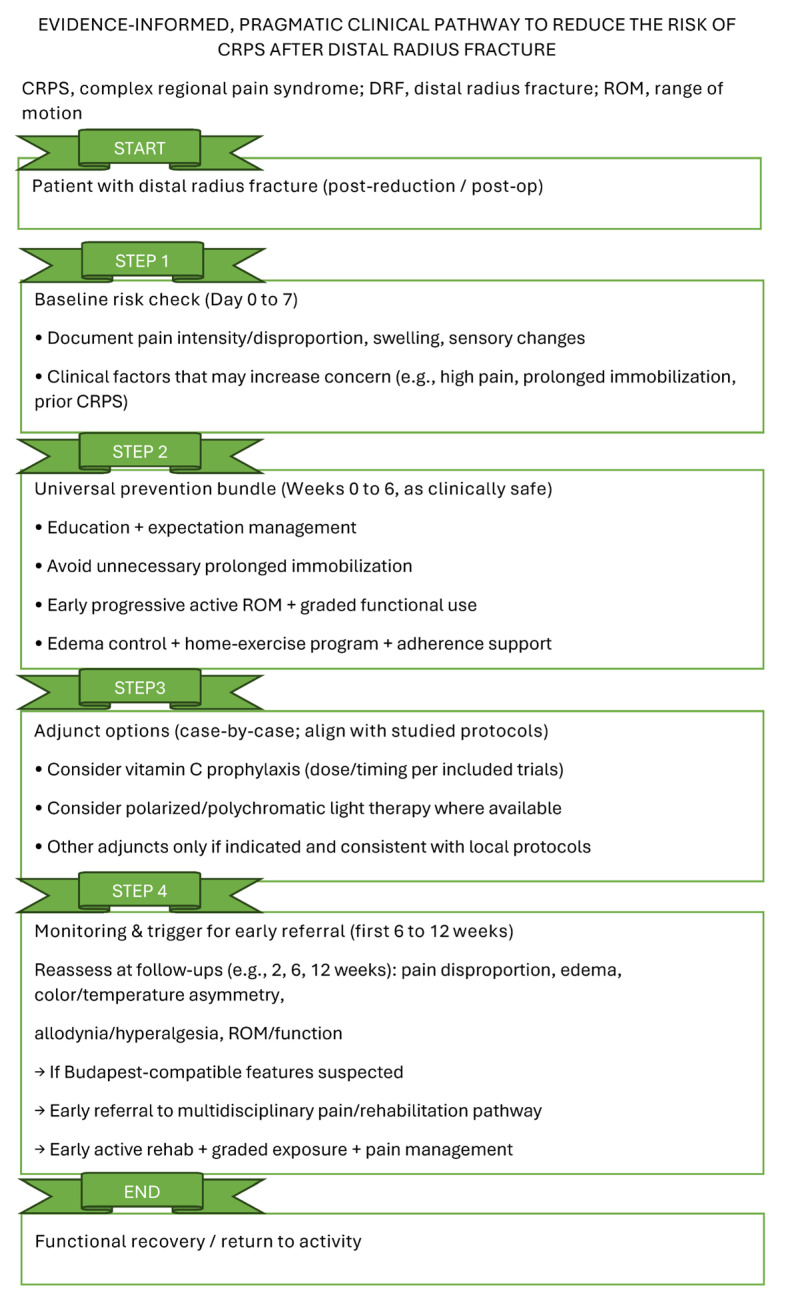
Clinical pathway to reduce CRPS risk after distal radius fracture (DRF), integrating baseline screening, universal prevention measures (education, graded activity, edema control and home exercise), optional adjuncts evaluated in the literature, and monitoring/referral triggers aligned with Budapest-compatible suspicion.

To facilitate implementation of the findings in routine care, we propose a pragmatic rehabilitation-oriented clinical pathway to reduce CRPS risk after distal radius fracture (Figure 4). This pathway integrates early baseline screening (days 0–7), universal preventive measures during the immobilization/early recovery phase (weeks 0–6), consideration of adjuncts on a case-by-case basis, and structured monitoring with clear referral triggers during weeks 6–12.

Nonetheless, important limitations must be considered when interpreting the findings. The overall number of included studies was limited, and heterogeneity in study design, diagnostic criteria, intervention protocols, and follow-up periods restricted the ability to compare results directly and prevented quantitative meta-analysis. Differences in immobilization practices, rehabilitation protocols, and surgical techniques across studies may have influenced CRPS incidence independently of the tested interventions. Several preventive strategies were evaluated in only one study each, which limits the certainty of conclusions regarding their effectiveness. Additionally, inconsistent use of validated diagnostic criteria across studies may have contributed to under- or overestimation of CRPS incidence. Using GRADE, the overall certainty of evidence ranged from low to very low across most interventions, mainly due to heterogeneity in CRPS diagnostic criteria, imprecision, and risk of bias in non-randomized studies.

Across the included studies, preventive approaches varied widely in content and intensity, which limits direct comparability but provides actionable components for rehabilitation pathways after DRF. From a practical standpoint, strategies that are low-cost and scalable—such as early progressive mobilization, structured home-exercise programs, and patient education—are particularly relevant for routine clinical settings. When pharmacological or nutritional prophylaxis is considered (e.g., vitamin C), clinicians should align dosing and timing with the protocols tested in the highest-quality studies and communicate the current certainty of evidence. Finally, implementing a standardized early-identification pathway for Budapest-compatible symptoms may reduce time to multidisciplinary treatment and mitigate functional impact.

## 5. Conclusions

The current evidence does not support a single standardized protocol for preventing CRPS after distal radius fracture. Early rehabilitation and progressive mobilization appear to be the most clinically promising strategies, although the available evidence remains limited. Vitamin C and light therapy may have potential roles, but their effects should be interpreted with caution because the evidence is inconsistent and, in the case of light therapy, based on a single study. Probiotics and aspirin have not shown convincing preventive effects. Further high-quality trials with standardized CRPS diagnostic criteria and clearly defined interventions are required.

## Figures and Tables

**Table 1:** Characteristics of included studies (design, sample, intervention/comparator, follow-up, outcomes, and diagnostic criteria for CRPS).

Authors	OCEBM—2011 Level	Study Design	No. Participants	No. CRPS	Mean Age	CRPS Diagnostic Criteria
Groenveld et al. (2022) [17]	3	Retrospective multicenter study	5488	20	48.8	Budapest
Boersma et al. (2022) [18]	4	Proof-of-concept study	129	12	59	Budapest
Sane et al. (2021) [12]	2	Open-label, prospective, randomized, parallel design study	144	27	57	Budapest
Eraghi et al. (2020) [19]	2	Double-blind, randomized controlled trial	103	15	60,27	Budapest
Alimian M. et al. (2021) [20]	2	Randomized, double-blind clinical trial	74	23	45	Veldman
Zlatkovic-Svenda et al. (2019) [21]	3	Prospective study	52	4	64	No
Boersma et al. (2018) [11]	3	Proof of concept cohort study	56	0	58	Budapest
Gillespie et al. (2016) [22]	4	Series of audits and service evaluations	490	25	unknown	Bruehl’s diagnostic criteria
Lei et al. (2016) [23]	2	Double-blind placebo controlled clinical trial	381	0	64	Atkins

CRPS, Complex Regional Pain Syndrome; OCEBM, Oxford Centre for Evidence-Based Medicine (Levels of Evidence, 2011) [28].

**Table 2:** Summary of preventive interventions and main findings for CRPS after distal radius fracture (DRF).

Authors	Duration Protocol	Preventive Intervention	Incidence CRPS (Study Group)	Secondary Results	Main Results
Groenveld et al. (2022) [17]	2014 to 2018	Shorter immobilization; differential diagnosis CRPS; informing the patient; active home exercise program; manage the psychological impact	0.36%	The annual incidence decreased from 23.2 (95% CI: 22.5–23.9) per 100,000 person years in 2014 to 16.1 (95% CI: 15.5–16.7) per 100,000 person years in 2018	The changing clinical approach towards CRPS, with focus on prevention and psychological aspects of posttraumatic pain, this decrease in CRPS incidence
Boersma et al. (2022) [18]	December 2012 to July 2017 Follow-up in weeks: After 8 to 12 weeks after the fracture	After immobilization an information leaflet with instructions for a home exercise program and explanation about of pain	0% (95% CI, 0.00–0.028)	2 patients were diagnosed with arthrosis, 5 with malunion, and 1 with carpal tunnel syndrome. In 4 patients, the pain was associated with stiffness in the hand and wrist.	Of the 129 patients included in this study, 12 reported disproportionate pain, and none were diagnosed with CRPS-I
Sane et al. (2021) [12]	January 2020 to March 2020 Follow-up in weeks: 12 weeks	Oral taking vitamin C (500 mg/day) + standard therapy (from fracture management). Administration of calcium (500 mg/day) and vitamin D (1000 IU/day) supplementation for 3 months during physiotherapy	11.3% (*p* = 0.023)—8 of 71	Gender, smoking, alcohol, and comorbidities were not found to be predictive factors for CRPS-I; fracture classification was significantly associated (*p* < 0.001). CRPS-I was diagnosed at an average of 66 ± 12 days after fracture management. The probability of no CRPS-I was higher and significant in the vitamin C plus standard group (88.7% vs. 74%, *p* = 0.020)	The prevalence of CRPS-I was 18.8% (27 of 144). The CRPS-I occurred in 11.3% of vitamin C plus Standard group participants (8 of 71), as against 26% (19 of 73) in standard group participants
Eraghi et al. (2020) [19]	From August 2016 to September 2017 Follow-up in weeks: 12 weeks	Oral taking 500 mg of aspirin daily for 7 days.	13.6% (*p* = 0.479)—6 of 44 patients	Lower rate of regional osteoporosis in the aspirin group (*p* = 0.047). 7 patients had pin tract infections (4 from aspirin group and 3 from placebo group, *p* = 0.708). Comminuted distal radius fractures more common in the patients with CRPS (*p* = 0.027)	The prevalence of CRPS in the aspirin group was lower (13.6%) than the placebo group (19.1%), but was not statistically significant
Alimian M. et al. (2021) [20]	April 2018 to October 2019 Follow-up in weeks: 12 weeks	Taking 500 mg vitamin C as a Bier block adjuvant	22.9% (*p* = 0.04)	Age, sex, time elapsed before surgery, and mechanism of trauma were not predictive of CRPS (*p* > 0.05)	The overall incidence of CRPS at follow-up visits in vitamin C group was significantly less than the controls (22.9% vs. 45.5%, *p* = 0.04)
Zlatkovic-Svenda et al. (2019) [21]	January 2014 to December 2017 Follow-up in weeks: 24 weeks after stop therapy	Bioptron (polarized, polychromatic, noncoherent, low-energy radiation) light + same protocol: nonsteroid anti-inflammatory drugs, exercises, and cryotherapy	0% (*p* < 0.05)	Light therapy accelerated pain relief (*p* < 0.05) and supination improvement (*p* < 0.05) at D15. The supination was significantly improved at D7 and D15 for the light therapy-treated group. CRPS occurrence was significantly reduced in the light therapy-treated group compared with the control group (*p* < 0.05), 0% and 15.4%, respectively. Complete hand fist-forming capacity was achieved in 16 control group patients (61.5%) and in 19 light therapy-treated group (73.1%), although this did not reach statistical significance (*p* = 0.375)	Light therapy significantly accelerated pain relief and improved supination in elderly patients, compared with conventional treatment alone
Boersma et al. (2018) [11]	December 2012 to November 2014 Follow-up in weeks: 12 weeks	Patients were instructed to perform specific exercises at home daily over a 6 weeks and to resume normal activities gradually without involvement of a physiotherapist	0% (95% CI of 0.00–6.38%)	--	9 patients (16%) scored positive on the subjective Budapest diagnostic criteria. None were diagnosed with CRPS-I when the objective symptoms of the Budapest criteria were assessed
Gillespie et al. (2016) [22]	2004 to 2013	Service audits and evaluations to introduce a patient information program, staff education, correct cast management and encouraging early light function in the cast	<1%	The incidence of CRPS after DRF dropped to 10% over the course of the study	The incidence can be reduced significantly with simple low-cost measures and attention to detail
Lei et al. (2016) [23]	November 2010 to March 2015 Follow-up in weeks: 36 weeks	To receive skimmed milk containing a commercial probiotic (Lactobacillus casei Shirota) daily for a period of 6 months after the fracture	Not applicable	No significant differences in DASH and CRPS scores between the groups. No differences between the treatment groups with regard to wrist range of movement. Patients treated with LcS exhibited significantly higher grip strength, at months 2 to 5, but not at the first and last months	LcS consumption could accelerate the healing process of distal radius fracture to a significant extent, at least during the first 4 months after the injury

CRPS, Complex Regional Pain Syndrome; CRPS-I Complex Regional Pain Syndrome Type I; CI, confidence interval; IU, international units; DRF, distal radius fracture; DASH, Disabilities of the Arm, Shoulder and Hand questionnaire.

## Data Availability

No new data were created or analyzed in this study. Data sharing is not applicable to this article.

## Supplementary Materials

The following supporting information can be downloaded at: https://www.mdpi.com/article/10.3390/jfmk11020158/s1, Table S1: PRISMA 2020 reporting summary and manuscript cross-referencing for this systematic review. Reference [37] is cited in the Supplementary Materials.
